# A facile and effective approach to prepare К-carrageenan / polyvinyl alcohol hydrogel as a potential wound dressing

**DOI:** 10.1038/s41598-026-62067-4

**Published:** 2026-07-19

**Authors:** Hesham M. Fahmy

**Affiliations:** Textile Research and Technology Institute, National Research Centre, Dokki, Giza Egypt

**Keywords:** К-carrageenan, Polyvinyl alcohol, Potassium tetraborate, Hydrogel, Non-woven cotton fabric, Wound dressing, Biochemistry, Biotechnology, Chemistry, Materials science

## Abstract

Since К-carrageenan (КC) can be crosslinked by potassium cations while polyvinyl alcohol **(**PVA) can be crosslinked by borated anions, the present work was undertaken to crosslink the КC/PVA blends in one step using potassium tetraborate tetrahydrate (PTB) as a crosslinker for forming КC/PVA/PTB hydrogels. Factors affecting crosslinking of that blends such as PTB concentration, steeping time in PTB aqueous solution, КC/PVA weight ratio, and crosslinker type were studied. The results indicated that the optimal conditions for preparing the КC/PVA/PTB gels are: КC/PVA weight ratio, 1; PTB concentration, 4%; and steeping time, 8 h. Among boric acid, potassium chloride, borax, and PTB as potential crosslinkers for the КC/PVA (50/50) matrix, the later, i.e. PTB, achieves the highest crosslinking magnitude and produces KC/PVA/PTB gel having a superior liquid-holding capacity (1020%) as well as high gel fraction (98.6%) properties. The chemical structure of the formed КC/PVA/PTB gel was confirmed using FTIR analysis. The potential application of the КC/PVA/PTB gel as wound dressings was investigated by treating non-woven cotton fabric samples with КC/PVA (50/50) matrices in absence and presence of curcumin or silver nano-particles (Ag-NPs) as bio-additives followed by crosslinking with PTB. The prepared dressings exhibits superior antibacterial activities as well as significant mechanical properties expressed in the good tensile strength, elongation at break, elastic modulus, stiffness, air permeability, and porosity properties. Besides, the treated fabric exhibits moderate cytotoxicity. The prepared Ag-NPs were evaluated via UV–Vis, TEM, and XRD analysis whereas the prepared dressings were characterized using SEM and EDX analysis.

## Introduction

Carrageenans are nontoxic water soluble sulfated polysaccharides extracted from the red marine algae belonging to the family Rhodophyceae^1–3^. Due to their thickening, suspending, and gelling properties, carrageenans are industrially used in the food, medical, ceramics, and coatings applications^4^. Carrageenans are named as mu-, kappa-, nu-, iota-, lambda-, theta-, and xi-carrageenans according to amount and position of sulfate groups that affect their chemical and functional properties^5^. K-carrageenan (КC) chemical structure is composed of linear chains having alternated (1–3)-linked a-galactose-4-sulfate and (1–4)-linked 3,6-β- anhydrogalactose^6–10^.

КC is occurred in heated aqueous solutions as random coils but upon lowering the solution temperature to a certain temperature, a conformational transition from the coils form to double helices occurs^11,12^ depending on the temperature, ionic strength, and concentration of that biopolymer^12,13^. The КC double helices are parallel strands in twisted structures and stabilized by hydrogen bonds. The КC sulfate groups (-OSO^−^ _3_) exist in outer of the double helix and have diverse affinities for ions of potassium, lithium, sodium, barium, zinc, and calcium^14–16^. КC sulfate groups form ionic bonds with K^+^ ions, as an example, in which K^+^ ion simultaneously interacts with two sulfate groups of an anhydrous galactose forming a three-dimensional network strong gel^7,12^. The strength of the КC gel is greatly governed by the КC concentration in addition to cation type and concentration^17–20^. Despite the carrageenan merits, its high hydrophilicity, low mechanical strength properties, and reactivity limit its utility^21,22^. Therefore, carrageenans may undergo chemical and physical modifications to overcome such limitations. In that regard, carrageenans are normally blended with other polymers such as poly (vinyl alcohol)^23^ polyvinyl pyrrolidone^24^ and alginates^25^ to improve their processability.

On the other hand, poly (vinyl alcohol) (PVA) is a liner water soluble synthetic polymer prepared by hydrolysis of polyvinyl acetate. The chemical and physical properties of PVA are strongly governed by the degree of polymerization and hydrolysis of its parent polyvinyl acetate^26–29^. The attractive properties of PVA such as the excellent film forming properties and solubility in water renders it to be used in the medical, textiles, paper, adhesives, etc. industries^28,29^. PVA is capable to form different hydrogel systems having the ability to retain large amounts of water, either by physical or chemical means. PVA can be crosslinked chemically by means of the aldehydes such as formaldehyde and glutaraldehyde, citic acid as a polycarboxylic acid, glycidyl methacrylate, titanium and cupric salts, chromates and vanadates, as well as boric acid and borates^30^. The later produces industrially important gels because of their attractive chemical and physical properties. The PVA-Borax reaction proceeds via mono-diol and di-diol complexes formation^31–33^. The PVA gelation using borates depends on the PVA aqueous solution concentration and temperature, PVA molecular weight and degree of hydrolysis, and cation-boron ratio^31^; the pH and the borates origin are not significant to PVA gelling^34^. At low ratios of borate anions, boron reacts with PVA as mono-diol in addition to a negligible number of di-diol complexes (crosslinks)^31^. Meanwhile, borax is a salt of a strong base and a weak acid which may be hydrolyzed in water solution forming a boric acid-borate buffer^35^.

Many studies reported the КC/PVA hydrogels preparation^36–42^. Croitoru et al. synthesized КC/PVA hydrogel physically using four successive freezing–thawing cycles for the КC/PVA blends^36^. Rasool et al. prepared КC/PVA hydrogels by crosslinking КC/PVA blends using КC/PVA using hydrogels (3-aminopropyl) triethoxysilane as a crosslinker^37^. Feng et al. synthesized a conducting КC/PVA hydrogel using borax as a crosslinker followed by KOH treatment^38^. Bajpai et al. prepared PVA/carrageenan composite films by crosslinking aqueous solutions of PVA and carrageenan with borax and K^+^ ions at room temperature^39^.

Actually, using of a single salt, rather than two salts, to crosslink КC/PVA blends offers economic and ecological benefits via producing a homogenous networking, reducing chemical waste and energy consumption, lowering toxicity, as well as minimizing the processing time^43^. The present research work was undertaken to prepare КC/PVA gels by crosslinking of КC/PVA blends in one step using potassium tetraborate tetrahydrate as a co-crosslinker for both the КC and PVA.

## Experimental

###  Materials

K-carrageenan (КC), supplied Sigma, was used. Commercial grade of polyvinyl alcohol (PVA) namely Hoe^®^ T 3713 (medium viscosity, alkali resistant, and containing acetyl groups), was kindly supplied by Hoechst, Germany. Commercial non-woven cotton fabric was supplied by a commercial company, Egypt. Potassium tetraborate tetrahydrate (PTB) and curcumin are supplied from Sigma and used. Boric acid, borax, potassium chloride, silver nitrate, tri-sodium citrate, and sodium hydroxide are of laboratory grade chemicals.

### Methods

####  Preparation of КC/PVA gel film

The КC and PVA solutions were prepared individually by dissolving 1 g of such polymers in 100 mL of distilled water at 75 °C with stirring till complete dissolution. Different formulations of КC/PVA blends were prepared by mixing different weights of the above mentioned solutions with stirring to obtain homogenous solutions of 1% net concentration which were then casted into a level Teflon-coated glass plates, and left to dry at 40 °C in air circulated oven. To obtain КC/PVA gels, the prepared films were immersed in specific concentration of PTB, borax, boric acid, or potassium chloride for a period of time.

#### Silver nanoparticles preparation

The Ag nano-particles (Ag-NPs) were synthesized according to the chemical reduction method^44,45^. Typically, 50 mL of 1 × 10^− 3^ M of AgNO_3_ was boiled and then 5 mL of 1% tri-sodium citrate was added drop wisely with stirring to that solution until the color was changed to pale yellow which confirms the formation of Ag-NPs.

####  Preparation of КC/PVA dressing containing Ag-NPs or curcumin

The КC/PVA dressings loaded with Ag-NPs or curcumin were prepared by padding non-woven cotton fabric samples in КC/PVA (50/50) blends solutions containing 1% Ag-NPs or 0.1% curcumin followed by drying at 80 °C/5 min, crosslinking with 4% PPB solution, and finally drying at 80 °C/5 min.

### Testing and analysis

- Statistical analysis: all the analyses in this manuscript were performed at least three times to ensure accuracy. The results obtained are the average of three samples with a standard deviation of less than 5%. In addition, the significance level, i.e. p, between the different groups was ≤ 0.05.
- The swelling degree (SW) of the prepared gel was evaluated by steeping of a specific weight of the prepared КC/PVA films in distilled water for 24 h followed by withdrawing it from the distilled water, removing the excess water by filter paper, and reweighing again. The percent DS was carried out to evaluate the absorbed water amount and determined using the following equation:

DS (%) = Ws − Wi/Wi × 100, where the Wi and Ws are the initial and wetted samples weights^7–9^.

- The swelling kinetics of the КC/PVA/PTB gel was evaluated using Peleg’s model according to the following Eqs.^46,47^:

$$S = S_{o} + \frac{t}{{k_{1} + k_{2} t}}$$

Where: S is the swelling at time t, S_0_ is the initial swelling at t = 0 which can be assumed to be equal zero for the dry gel, k_1_ is the kinetic constant of the model, and k_2_ is the Peleg capacity constant.

- The gel fraction (GF) of the prepared gel was performed to indicate the firmness degree of the prepared film. The percent gel fraction was estimated using the following equation: Gel fraction (%) = (Wf/Wi) × 100, where the Wi and Wf is the initial weight of the film sample and Wf is the constant dry weight of the swelled film sample^7–9^.
- The tensile strength (TS), elongation at break (EL), and elastic modulus of the prepared dressings were evaluated conferring to ASTM standard way D5035.
- The air permeability (AP) of the prepared dressings was assessed according to ATSM (D 737).
- Porosity and pore size of the treated non-woven fabric were evaluated using the Quantachrome instrument.
- The stiffness (S) of the prepared dressings was assessed due to JIS L1018.
- The antimicrobial activities of treated fabrics have been studied using colony forming technique (CFU) against the following bacteria strains^45,48,49^:

Gram-positive bacteria: *Staphylococcus aureus* (SA) (ATCC 6538-P).

Gram-negative bacteria: *Escherichia coli* (EC) (ATCC 25933).

- Cytotoxic studies.

- Cell lines and cell culture
  The human neonatal primary normal fibroblast (HDFn) cell line was purchased from the American Type Culture Collection (ATCC, USA). It was routinely cultured in the Basal Medium Eagle. The full medium consists of 10% fetal bovine serum (FBS) and 2 mM L-glutamine, in addition to a 1% antibiotic-antimycotic cocktail. All reagents were from Biowest (Nuaillé, France). Cells were maintained at sub-confluency at 37 °C in humidified air containing 5% CO_2_. For sub-culturing, monolayer cells were harvested after trypsin/EDTA treatment at 37 °C. Cells were used when confluence had reached 75%.
- The Cytotoxic assay (MTT)
  The MTT colorimetric assay was conducted according to the original procedure proposed by Hansen et al.^50^. The MTT (3-[4,5-dimethylthiazole-2-yl]-2,5-diphenyltetrazolium bromide), obtained from Merck KGaA (Darmstadt, Germany), was used to assess the cytotoxicity of the tested samples. It is based on the capability of the active mitochondrial dehydrogenase enzyme in living cells to cleave the tetrazolium rings of the yellow MTT and form dark blue insoluble formazan crystals. The crystals are solubilized, resulting in a dark blue color that is directly proportional to the number of live cells. Briefly, Cells (1 × 10^4^ cells/well) were seeded in serum-free medium in a flat-bottom 96-well microplate and treated with a slice of each sample (5 mm x 5 mm), repeated for 6 times for 24 h at 37 °C in a humidified 5% CO_2_ atmosphere. After incubation, the media and slices were carefully removed, and 40 µl of MTT solution / well was added and incubated for an additional 4 hrs. The MTT crystals were solubilized by adding 180 µl of acidified isopropanol / well. The plates were shaken at room temperature, followed by photometric determination of absorbance (OD) at 570 nm using a microplate ELISA reader (FLUOstar OPTIMA, BMG LABTECH GmbH, Ortenberg, Germany). Six replicates were performed for each concentration, and the average was calculated. The formula used to calculate the cytotoxicity was as follows: Percentage of relative viability = $\:OD\frac{sample}{Control}\:$X 100.

- The scanning electron microscope images (SEM) of the films samples were obtained using SEM Model Quanta 250 FEG (Field Emission Gun) attached with EDX Unit (Energy Dispersive X-Ray Analysis), with accelerating voltage 30 kV, magnification 14× up to 1,000,000 and resolution for Gun, FEI company, Netherlands.
- The Ag-NPs formation was confirmed by ultraviolet-visible (UV–Vis) spectroscopy in the range of 420–450 nm due to the Surface Plasmon Resonance band of the Ag-NPs using T80 spectrophotometer.
- The particles size of the Ag-NPs were obtained by trans-mission electron microscope (TEM) using a JEOL, JEM 2100 Felectron microscope at 200 kV.
- The X-ray diffraction (XRD) of the Ag-NPs was performed using Bruker D8 instrument.

## Results and discussion

**Fig. 1:**
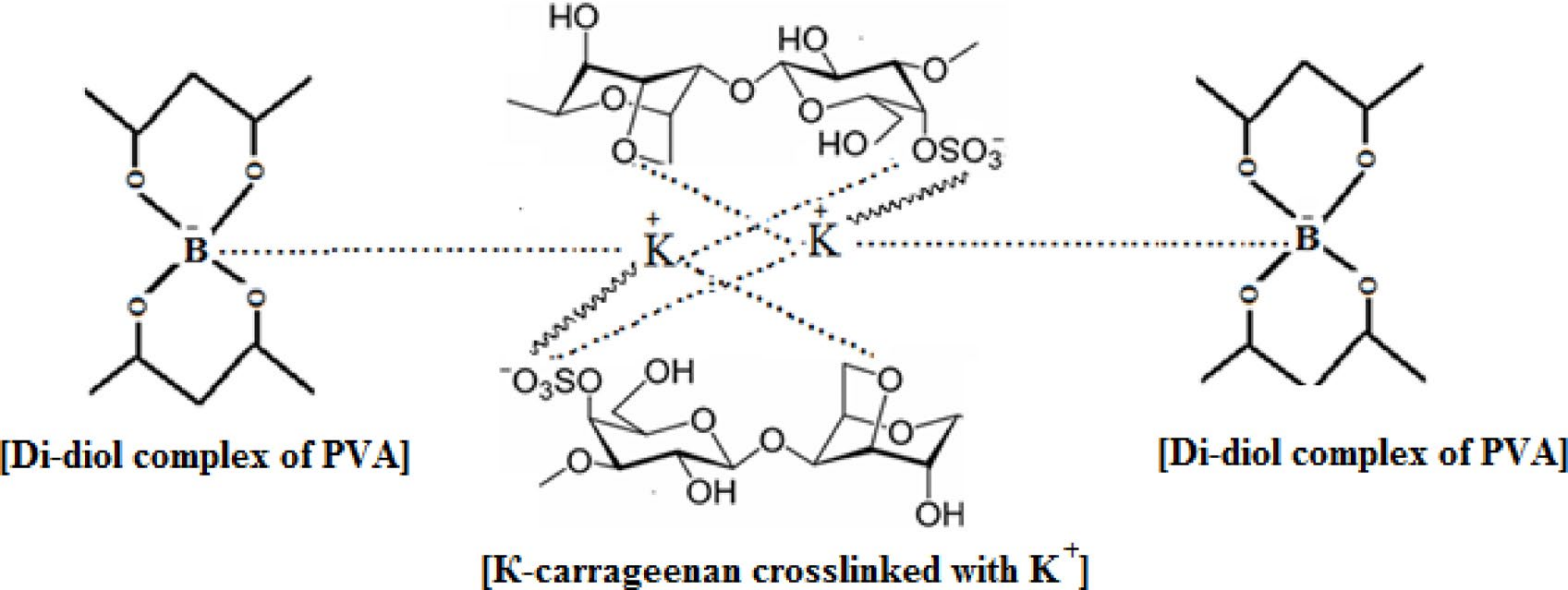
A proposed mechanism for crosslinking of КC/PVA blend with PTB.

It was reported that К-carrageenan (КC) can be gelled by K^+^ cations while polyvinyl alcohol (PVA) can be gelled using the borated anions. In that research work PTB was used to crosslink КC/PVA different blends according to the following proposed mechanism represented by Fig. 1:

Factors affecting crosslinking of КC/PVA matrices with PTB such as PTB concentration, steeping time, КC/PVA weight ratio, and the crosslinker type were studied along with an appropriate discussion.

### Factors affecting crosslinking of КC/PVA matrix with PTB

####  Effect of PTB concentration

**Fig. 2:**
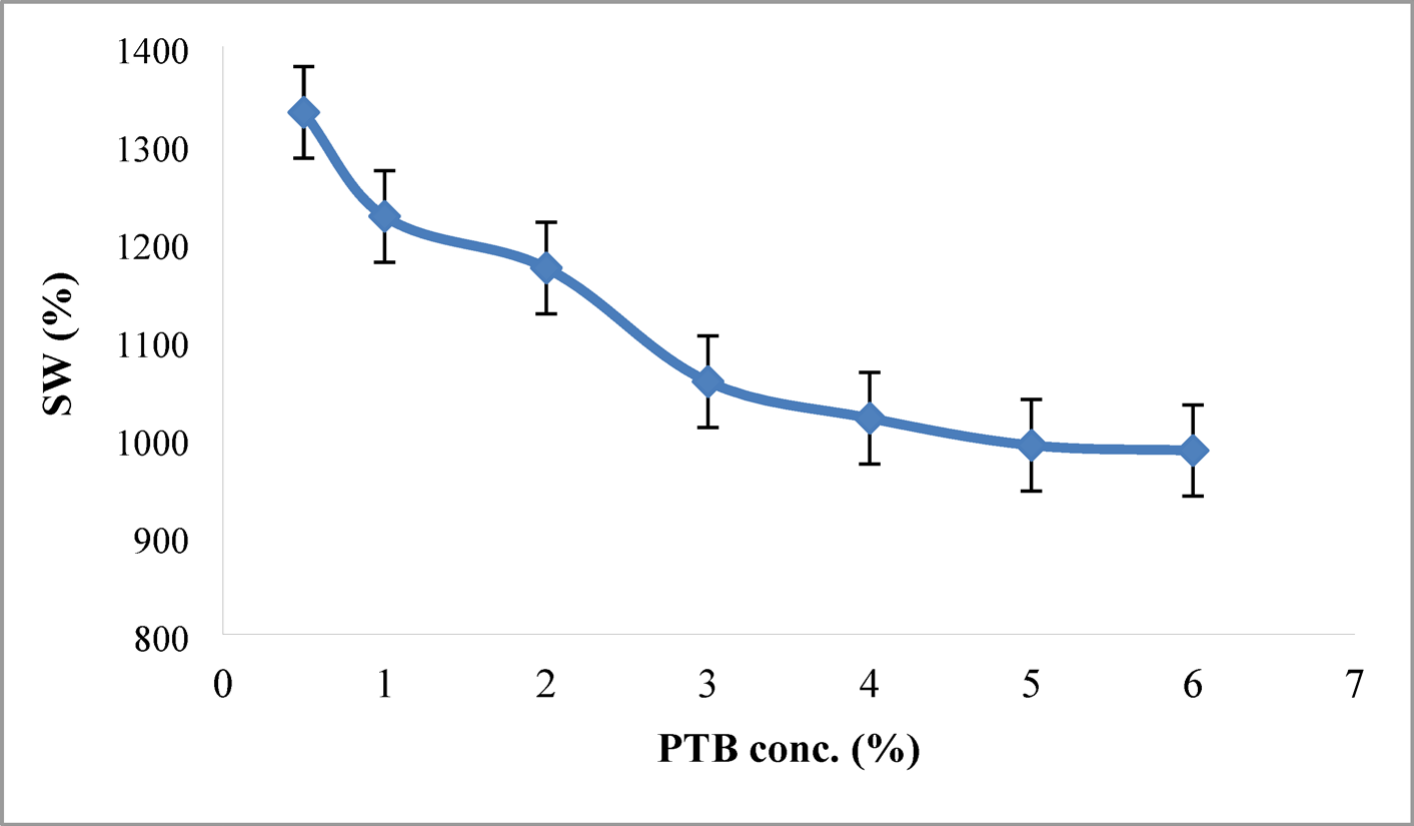
Effect of the PTB concentration on percent swelling of КC/PVA/PTB gel. КC/PVA weight ratio, 1; steeping time, 24 h.

**Fig. 3:**
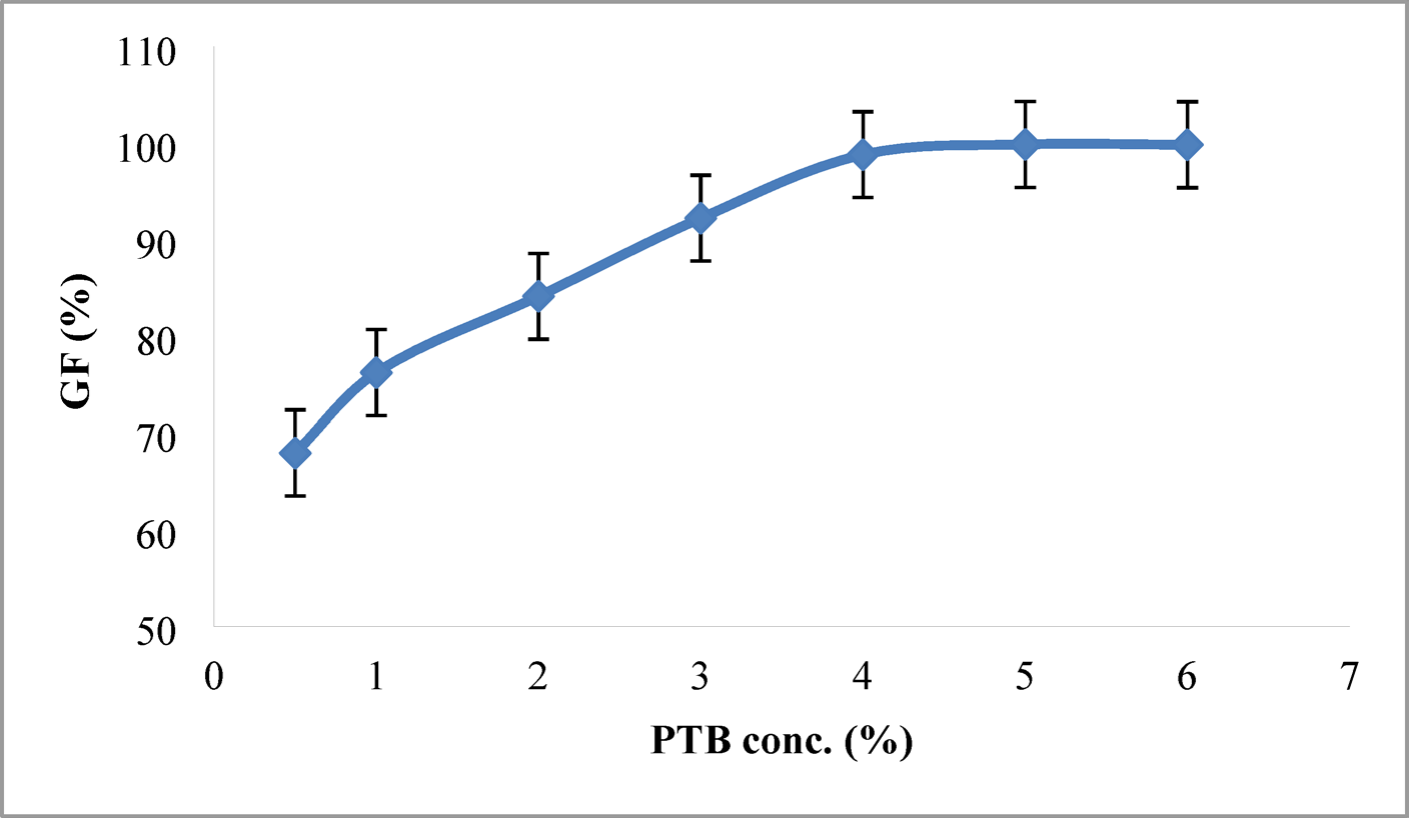
Effect of PTB concentration on percent gel fraction of КC/PVA/PTB gel. КC/PVA weight ratio, 1; steeping time, 24 h.

Figures 2 and 3 represent the impact of PTB concentration on percent swelling and gel fraction properties, respectively, of the КC/PVA/PTB formed gel. It is clear that increasing of the PTB concentration from 0.5 to 4% is accompanied by a gradual decreasing in swellability along with a progressive enhancement in gel fraction of the formed gel. The matter that can be explained by increasing of K^+^ cations capable to form ionic bonds with the sulfate groups of КC chains to form КC gel^7,8,10^ as well as availability of the [B_4_O_7_(OH)_4_]^2+^ anions that can interact with the PVA chains to form PVA gel^31–33^; all in state of entanglement that ultimately though gives rise to the formation of a firm КC/PVA/PTB gel. The further increasing in PTB concentration, i.e. up to 6%, causes a marginal reduction in magnitudes of such properties of the formed gel.

#### КC/PVA weight ratio

**Fig. 4:**
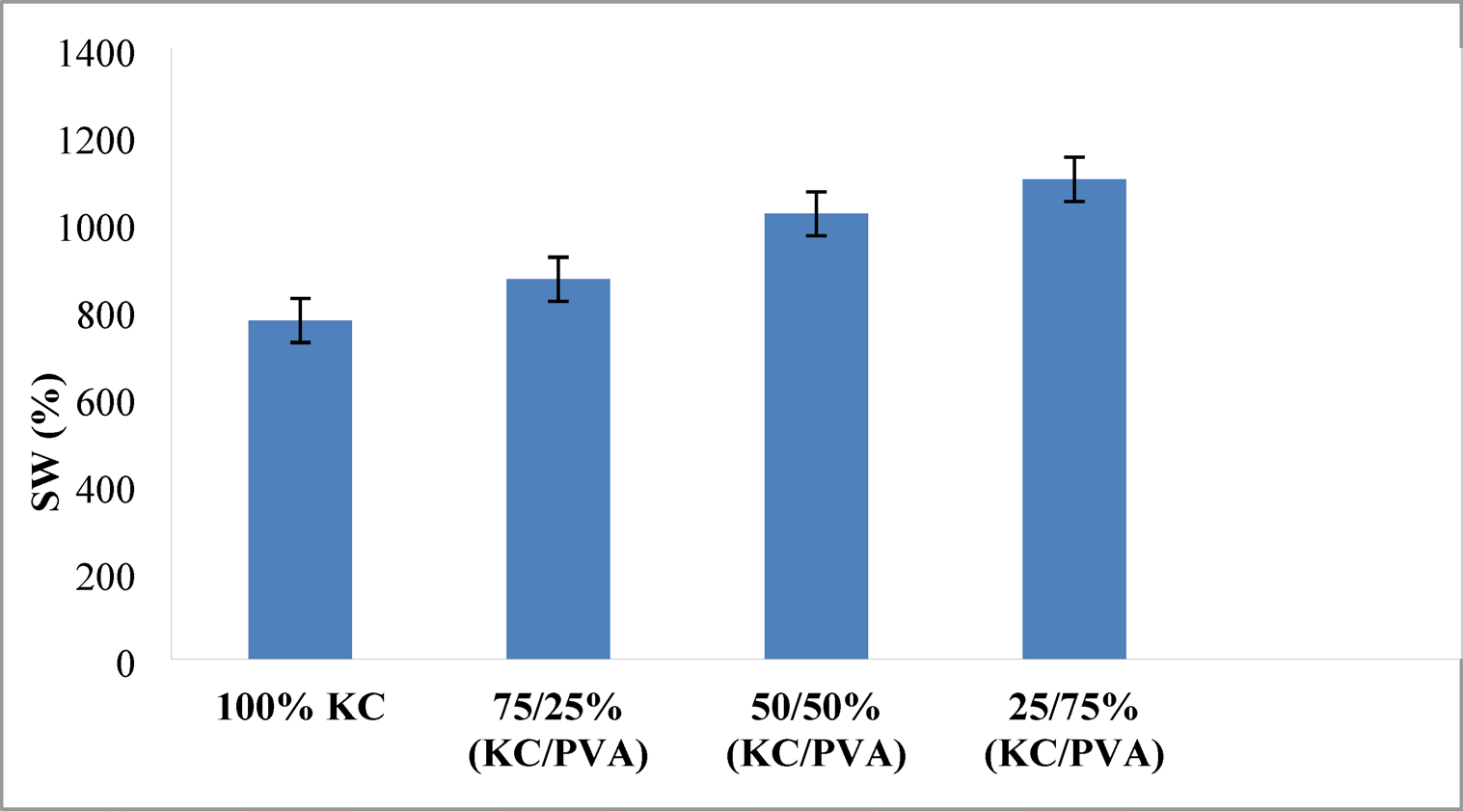
Effect of КC/PVA weight ratio on percent swelling of the КC/PVA/PTB gel. Steeping time, 24 h; PTB, 4%.

**Fig. 5:**
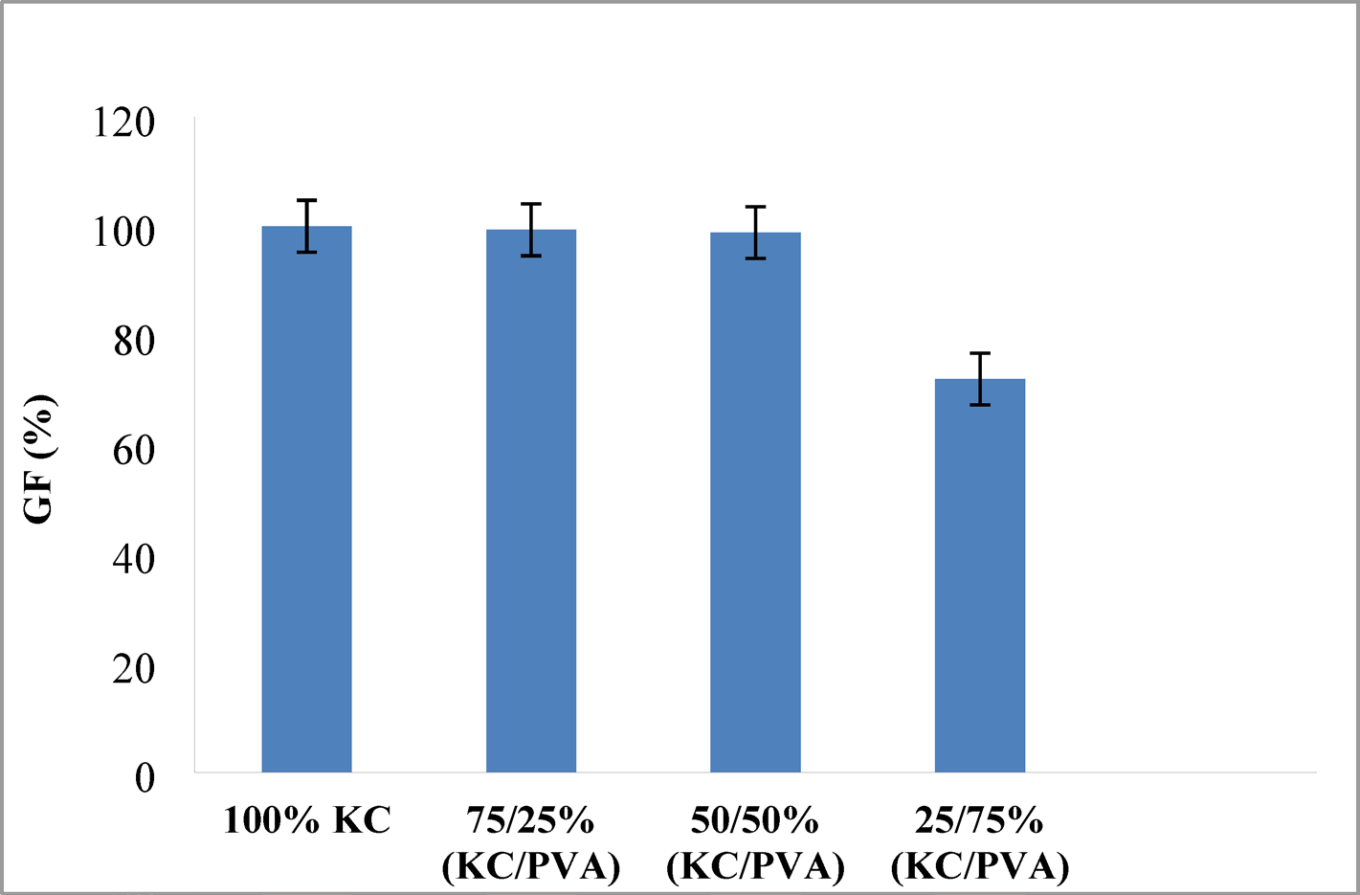
Effect of КC/PVA weight ratio on percent gel fraction of the КC/PVA/PTB gel. Steeping time, 24 h; PTB, 4%.

Figures 4 and 5 illustrate the effect of КC/PVA weight ratio on magnitudes of percent swelling and gel fraction properties of the formed КC/PVA/PTB gels. It is obvious that decreasing of КC weight ratio in the КC/PVA matrices from 100 to 75% brings about an increasing in extent of the swellability as well as a negligible reduction in extent of gel fraction of the formed gel. At ratio 50% of КC, the percent swelling significantly increases while the gel fraction of the formed gel slightly decreases reflecting increasing of the PVA molecules inside that blend matrix, the matter that impedes the collisions among КC molecules and K^+^ ions giving rise to the decreasing in extent of КC crosslinking. At 25% of КC, the gel fraction of the formed gel greatly falls down to reach 72% but the percent swelling slightly increases which may be ascribed to the reaction of PVA molecules with the B_4_O_7_^2-^ anions at that concentration of PTB to form mono-diol rather than di-diol complexes causing the lowering in extent of PVA crosslinking^29^. It seems that at the ratios 75 and 50% of КC in the aforementioned matrices, the formed КC/K^+^ gels include the PVA chains, reacted or unreacted, within their structures; all in state of entanglement and bound together through intermolecular H-bonds. Moreover, at the ratio 25% of KC, the K^+^ ions are present in plenty, as these ions crosslink only the KC molecules, which causes breaking of the intermolecular H-bonds inside the formed KC/PVA/PTB gel and hence the worse drop in gel fraction of that gel.

#### Effect of steeping time

**Fig. 6:**
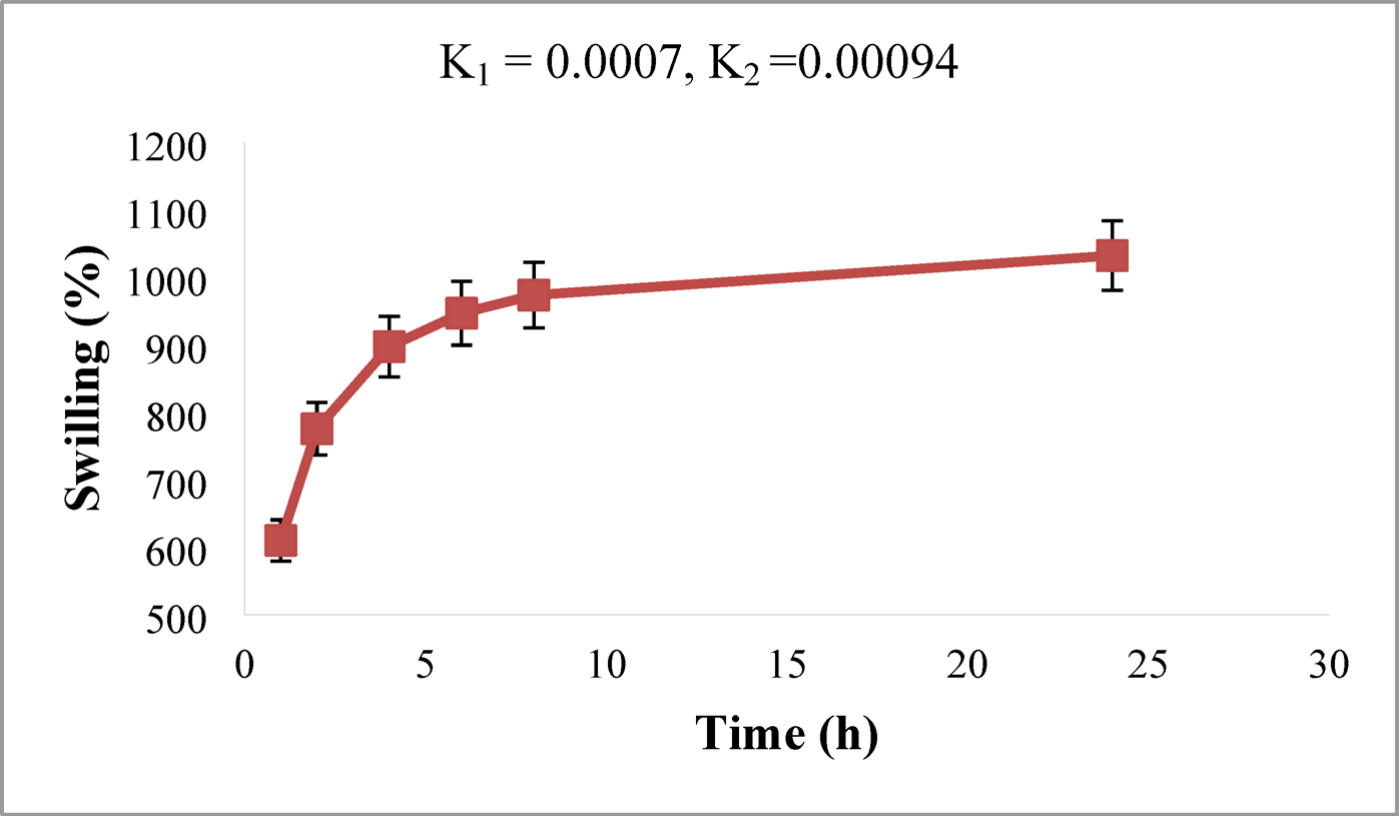
Swelling kinetics of the KC/PVA/PTB gel using the nonlinear Peleg’s model. KC/PVA weight ratio, 1; PTB concentration, 4%.

**Fig. 7:**
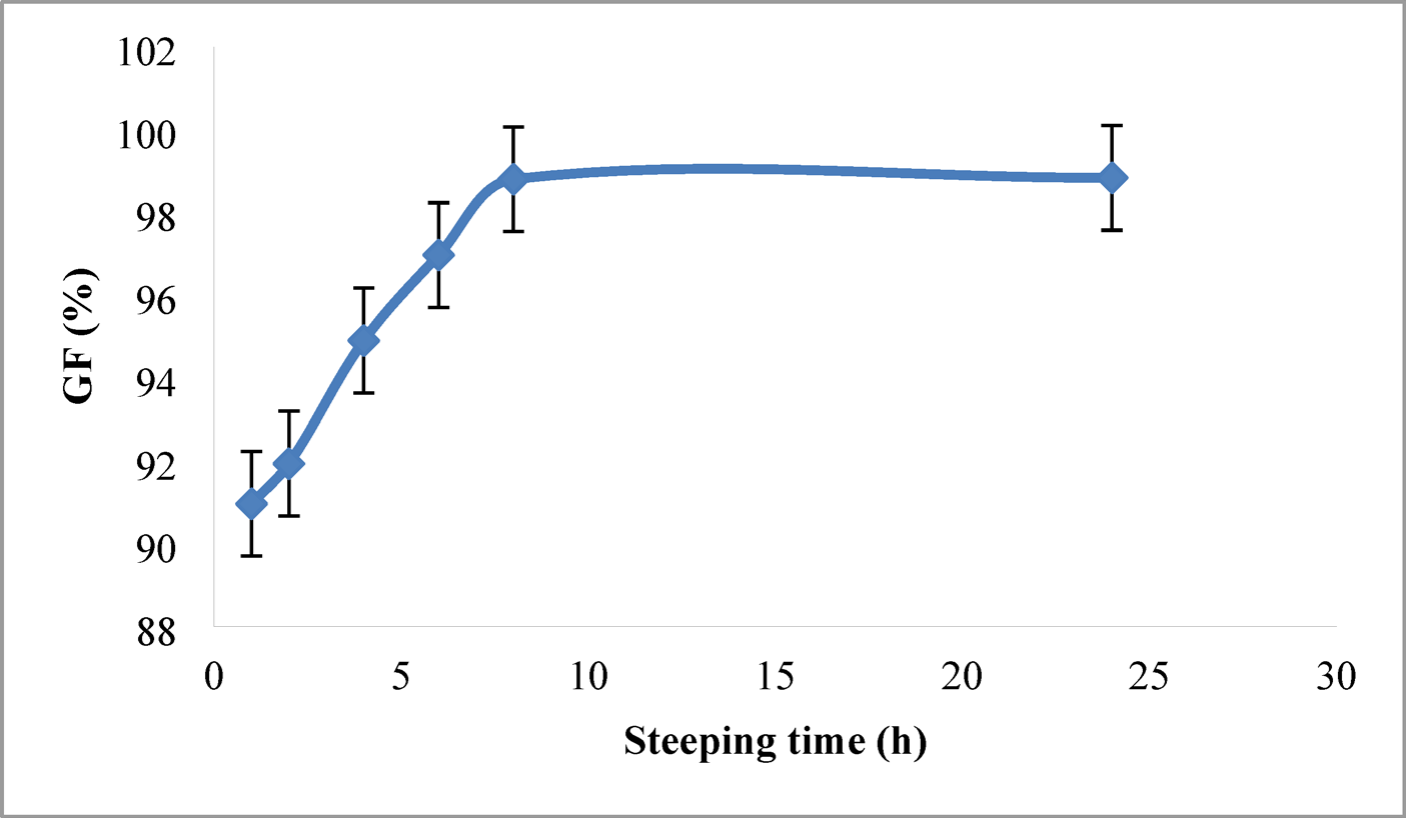
Effect of steeping time on percent gel fraction of the КC/PVA/PTB gel. KC/PVA weight ratio, 1; PTB concentration, 4%.

The steeping time of the KC/PVA matrix in 4% PTB aqueous solution affects the swelling and gel fraction extents of the formed KC/PVA/PTB gel. Figure 6 shows the kinetic curve of the КC/PVA/PTB gel swelling according to Peleg’s model^46,47^. It is obvious that the prolonged steeping of the KC/PVA matrix in 4% PTB for 24 h induces crosslinking of that matrix and optimizing the network density of КC/PVA/PTB gel resulting in a rapid initial swelling followed by a slower swelling rate till an equilibrium state. This can be explained by the fast rate of the КC/PVA/PTB gel formation and the subsequent fast water diffusion into the formed gel network which then after 8 h marginally decreases with increasing the steeping time till equilibrium state. On the other hand, Fig. 7 shows gel fraction of the formed КC/PVA/PTB gel as a function in steeping time. It is clearly seen that the percent gel fraction of the КC/PVA/PTB gel was progressively enhanced by the prolonging steeping of the КC/PVA matrix in PTB up to 8 h reflecting increasing of the chemical interactions among K^+^ ions and -OSO_3_^−^ groups of КC as well as PVA hydroxyl groups with B_4_O_7_^2−^ anions which in the net optimizes the network density of the КC/PVA/PTB gel. The further steeping time, i.e. for 24 h, causes a marginal decreasing in gel fraction extent suggesting attaining an equilibrium state with a probable structural relaxation and/or dissolution of loosely bound polymers species^48,49^. It seems that steeping of the КC/PVA matrix in 4% PTB for 8 h is the appropriate conditions to prepare the КC/PVA/PTB gel having a swelling degree of 1020% and a gel fraction of 98.8%.

#### Crosslinker type

**Fig. 8:**
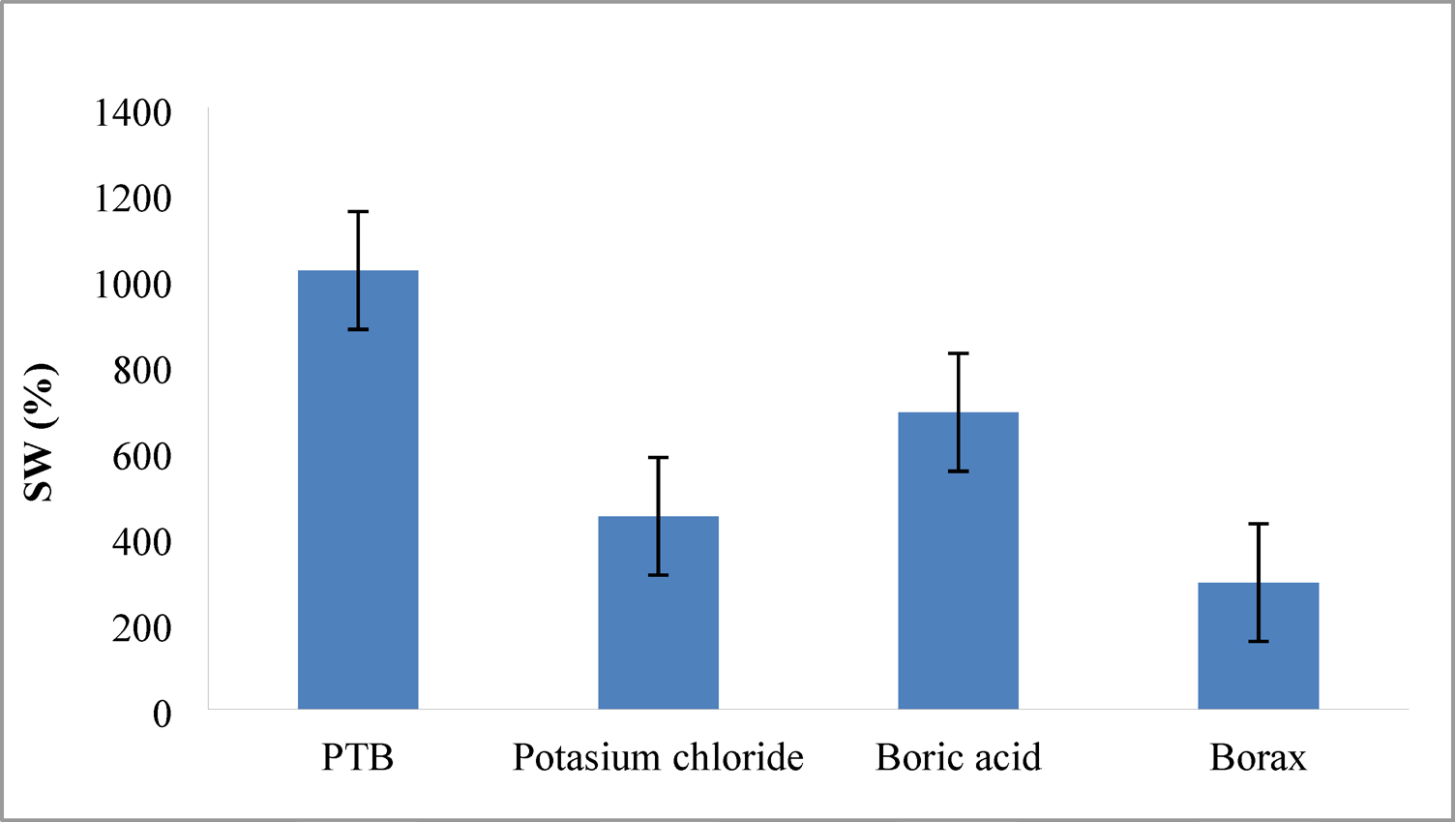
Effect of the crosslinker type on swellability of the КC/PVA/PTB gel. KC/PVA weight ratio, 1; salt concentration, 4%; Steeping time, 8 h.

**Fig. 9:**
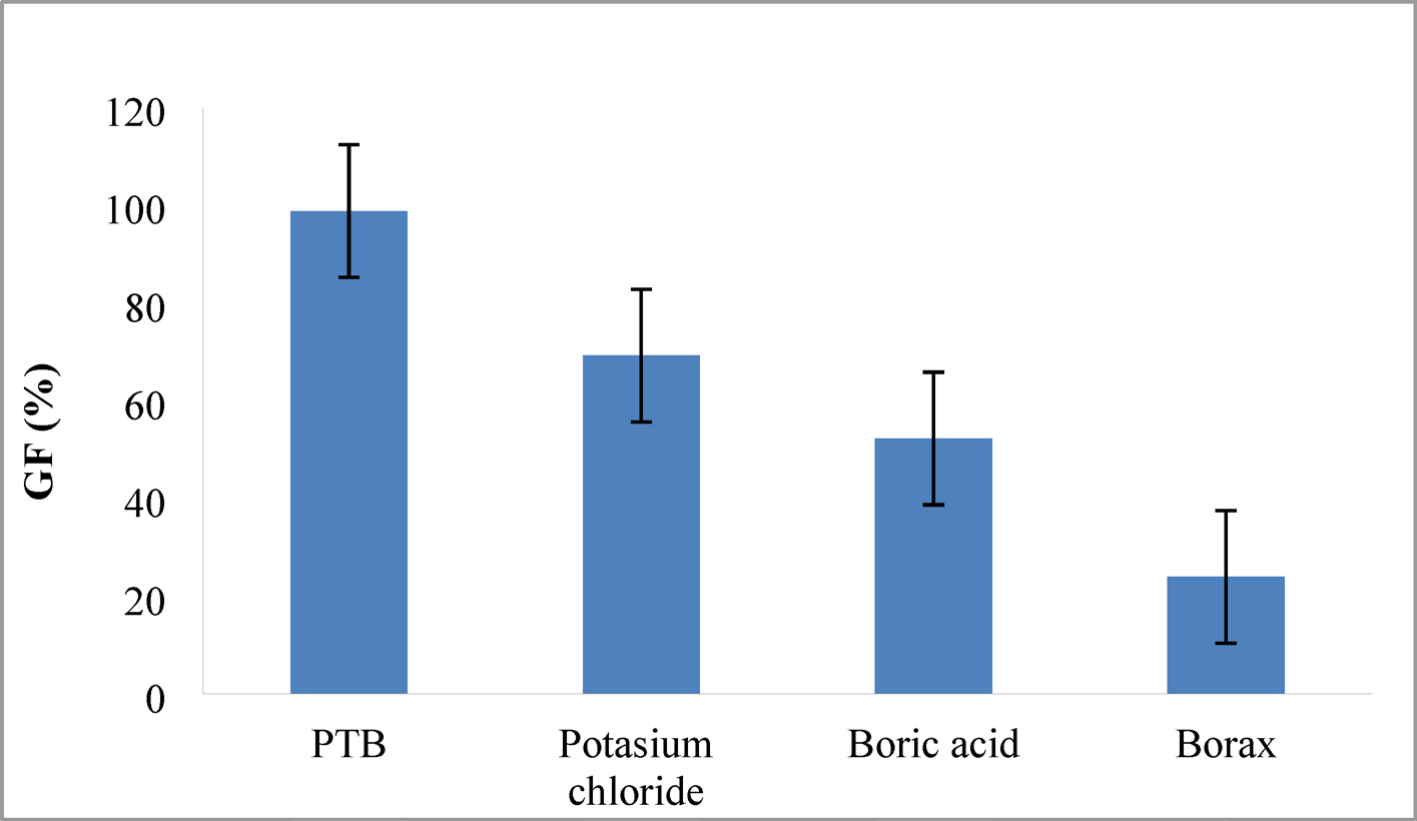
Effect of the crosslinker type on percent gel fraction of the КC/PVA/PTB gel. КC/PVA weight ratio, 1; salt concentration, 4%; Steeping time, 8 h.

Selecting the proper crosslinker type is highly important to enhance crosslinking extent of the КC/PVA matrix. Figures 8 and 9 represent the impact of crosslinker type on swelling and gel fraction properties, respectively, of the crosslinked КC/PVA matrix. It is clear that: (i) among the aforementioned crosslinkers, the КC/PVA matrix crosslinked with PTB has the highest percent swelling as well as gel fraction properties, and (ii) according to the crosslinker type, swellability of the crosslinked КC/PVA matrix can be arranged in the following order: PTB > boric acid > potassium chloride > borax, whereas the gel fraction of such crosslinked matrix can be arranged as follows: PTB > potassium chloride > boric acid > borax. The alteration in extents of the aforementioned properties of the crosslinked КC/PVA matrix is strongly related to the differences among that crosslinkers with respect to the metal type, nature of anion, rate of dissociation and/or decomposition, extent of crosslinking reactions and/or interactions, as well as location and distribution of the crosslinks^48,49,51,52^. Moreover, the KC/PVA matrix crosslinked with the boric acid has higher swelling and gel fraction magnitudes compared to that crosslinked with borax which may be attributed to the formation of strong hydrogen bonds inside the КC/PVA/boric acid gel structure between boric acid and hydroxyl groups of the КC molecules^53^.

### Characterization of the prepared КC/PVA/PTB hydrogel and silver nano-particles

#### FTIR

**Fig. 10:**
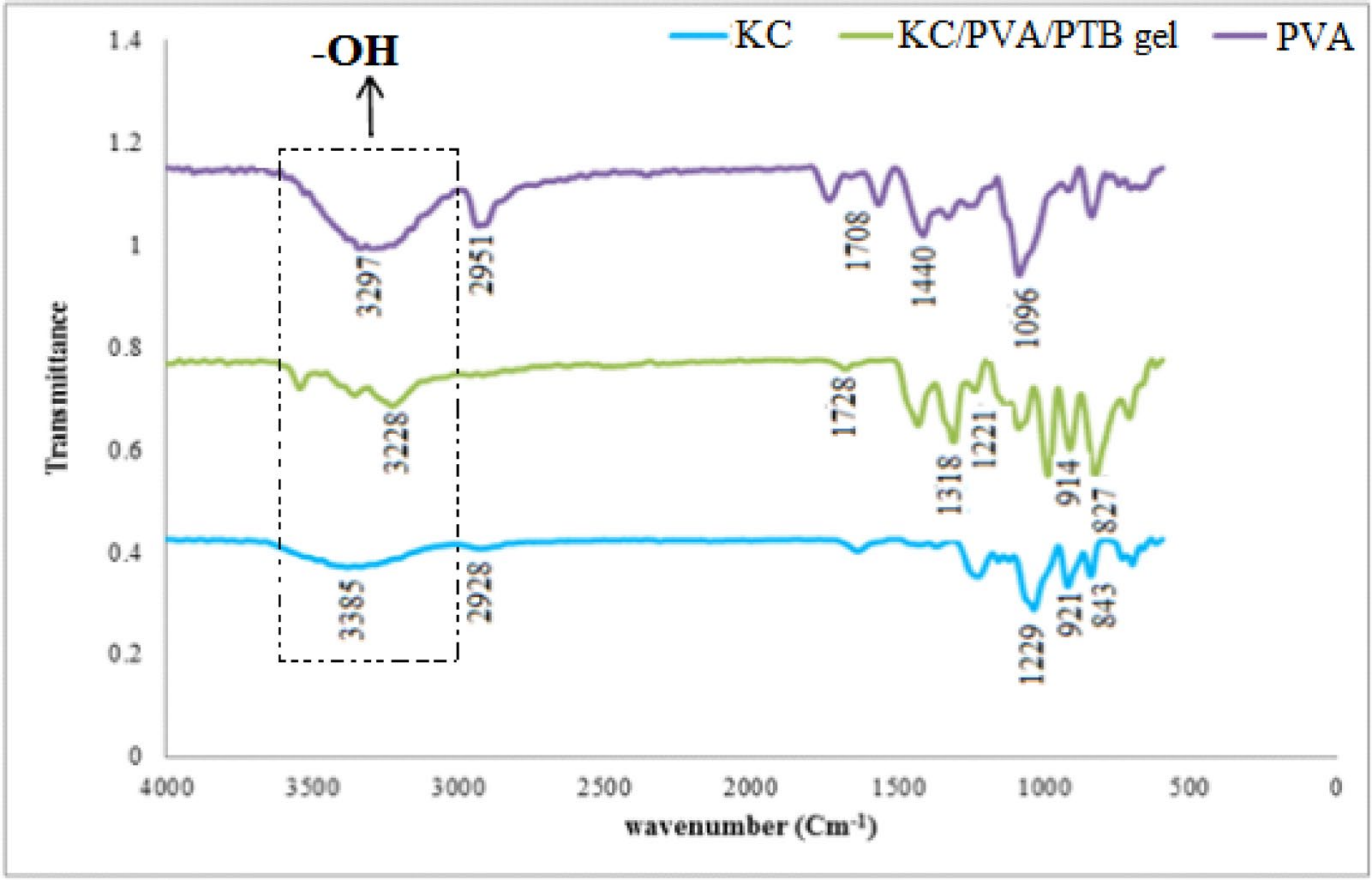
FTIR spectra of PVA, КC, and КC/PVA/PTB film.

The FTIR spectra of PVA, КC, and КC/PVA/PTB gel are represented by Fig. 10 which includes:

- Peaks belonging to PVA spectrum: a broad peak at 3365 cm^− 1^ assigned to OH group, two bands at 2951 due to asymmetric stretching of CH_2_ and 2928 cm^− 1^ corresponding to symmetric stretching vibrations of CH_2_ groups, a band at 1708 cm^− 1^ corresponding to C = O of the acetyl group, since the PVA is a partially hydrolyzed, a peak at 1440 cm^− 1^ due to –CH_2_ groups, a band at 1141 cm^− 1^ corresponding to C–O (crystallinity e sequence of PVA), and a band at 1096 cm^− 1^ due to stretching of C = O^54^.
- Peaks belonging to КC spectrum: a broad peak at 3414 cm^− 1^ assigned to OH stretching vibrations, a band at 2928 cm^− 1^ corresponding to CH_2_ groups, 1700 cm^− 1^ corresponding to carboxylic group, and three bands at 843, 921 and 1229 cm^− 1^ assigned to the d-galactose-4-sulfate, 3,6-anhydridegalactose as well as ester sulfate stretching vibrations respectively^7–10^.
- Peaks belonging to the КC/PVA/PTB gel spectrum: a narrow peak at 3408 cm^− 1^ corresponding to -OH groups; this peak is slightly shifted and exhibits much lower intensity compared to that of PVA and КC. The intensities percent of -OH group peaks of KC, PVA, and the КC/PVA/PTB gel can be expressed as 13.1/83.3/3.6% respectively, suggesting the consuming of some of the PVA hydroxyl groups in formation of diol complexes (Fig. 4) inside the gel structure. In addition, the other peaks are a peak at 3228 cm^− 1^ assigned to hydrogen bonding -OH groups, a band at 1728 cm^− 1^ corresponding to C = O of PVA acetyl groups, three bands at 827, 914 and 1226 cm^− 1^ assigned to the d-galactose-4-sulfate, 3,6-anhydridegalactose as well as ester sulfate stretching vibrations respectively of КC, and a peak at 1318 cm^− 1^ due to asymmetric stretching vibration of four coordinate boron to oxygen [(B_(4)_–O)] of the crosslinker PTB^55^, the matter that confirms formation of the КC/PVA/PTB gel.

### Characterization of the synthesized Ag-NPs

#### UV–Vis absorption spectra of the synthesized Ag-NPs

**Fig. 11:**
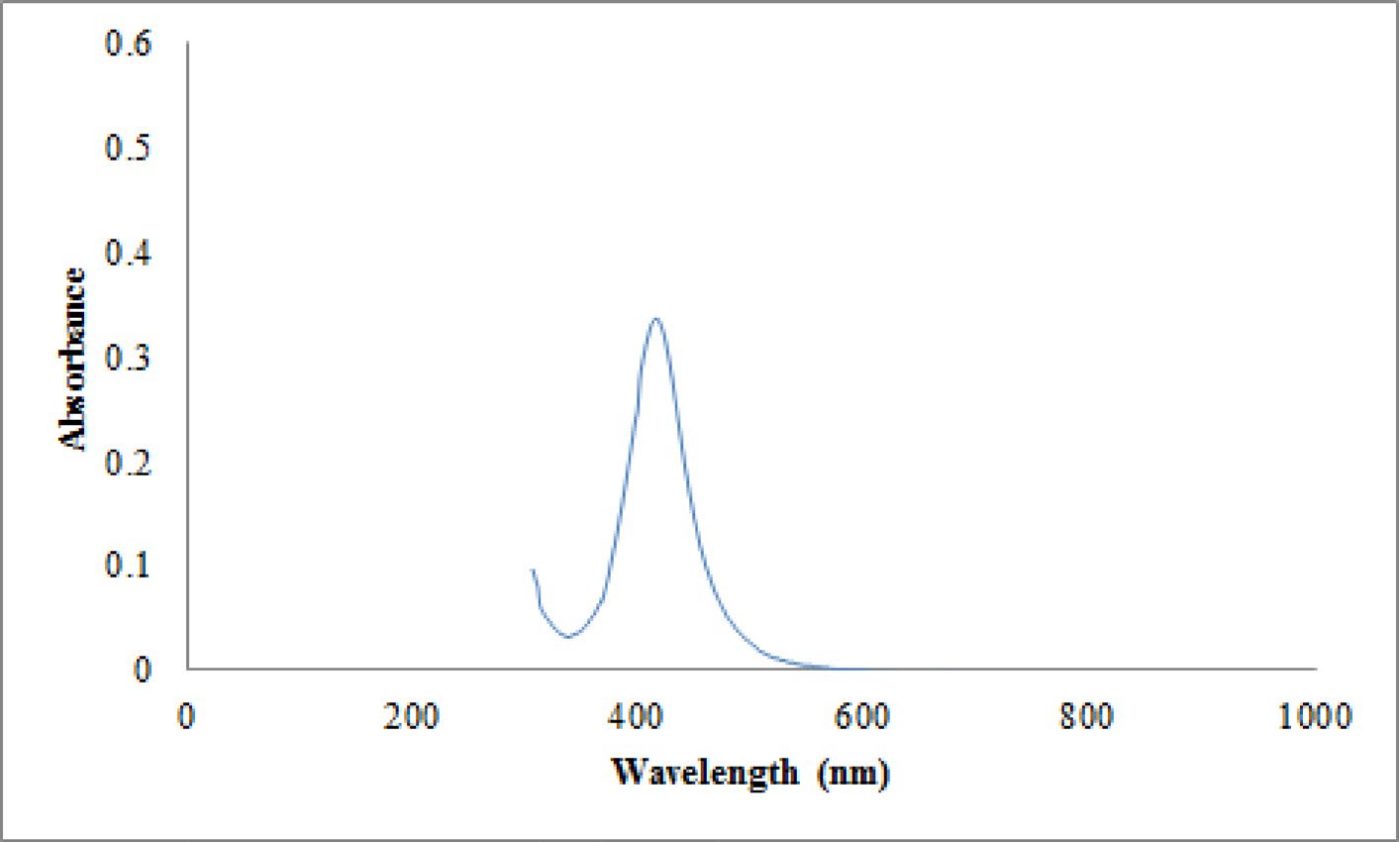
UV–Vis absorption spectra of the synthesized Ag-NPs.

Figure 11 shows the UV-Vis absorption spectra of the synthesized Ag-NPs colloidal solutions. It is clear that, the maxima absorption peak of Ag-NPs is at 430 nm, the matter that confirmed the successful reduction of Ag^+^ to Ag^0^ and formation of Ag-NPs using tri-sodium citrate as a reductant. Moreover, the narrow absorption peak indicates the low size distribution of the synthesized Ag-NPs^56^.

####  TEM analysis

**Fig. 12:**
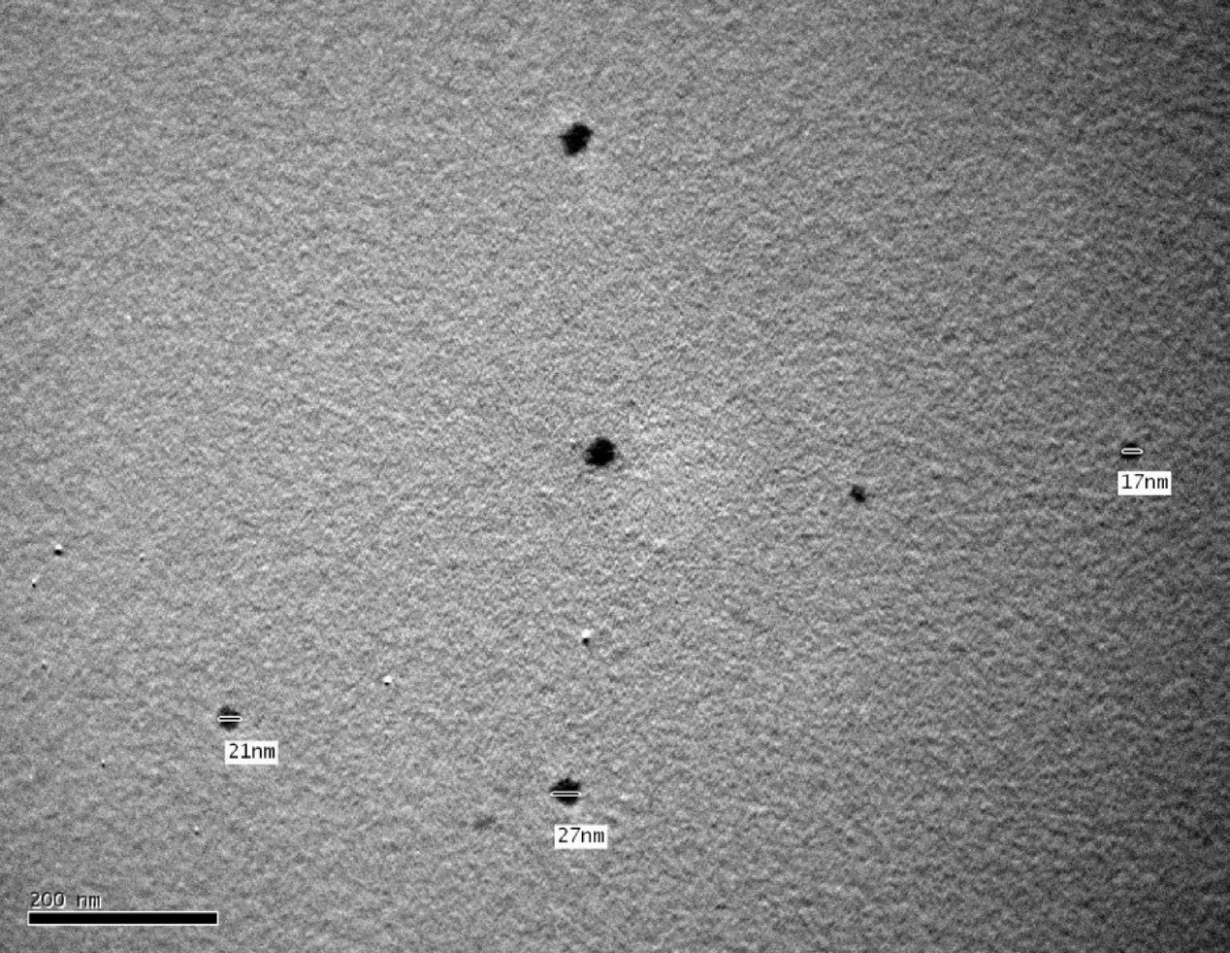
TEM image of the prepared Ag-NPs.

Figure 12 shows the TEM image of the prepared Ag-NPs. It is obviously seen that the prepared Ag-NPs have a roundish shape and sizes approximately below 21 nm.

#### X-ray diffraction (XRD)

**Fig. 13:**
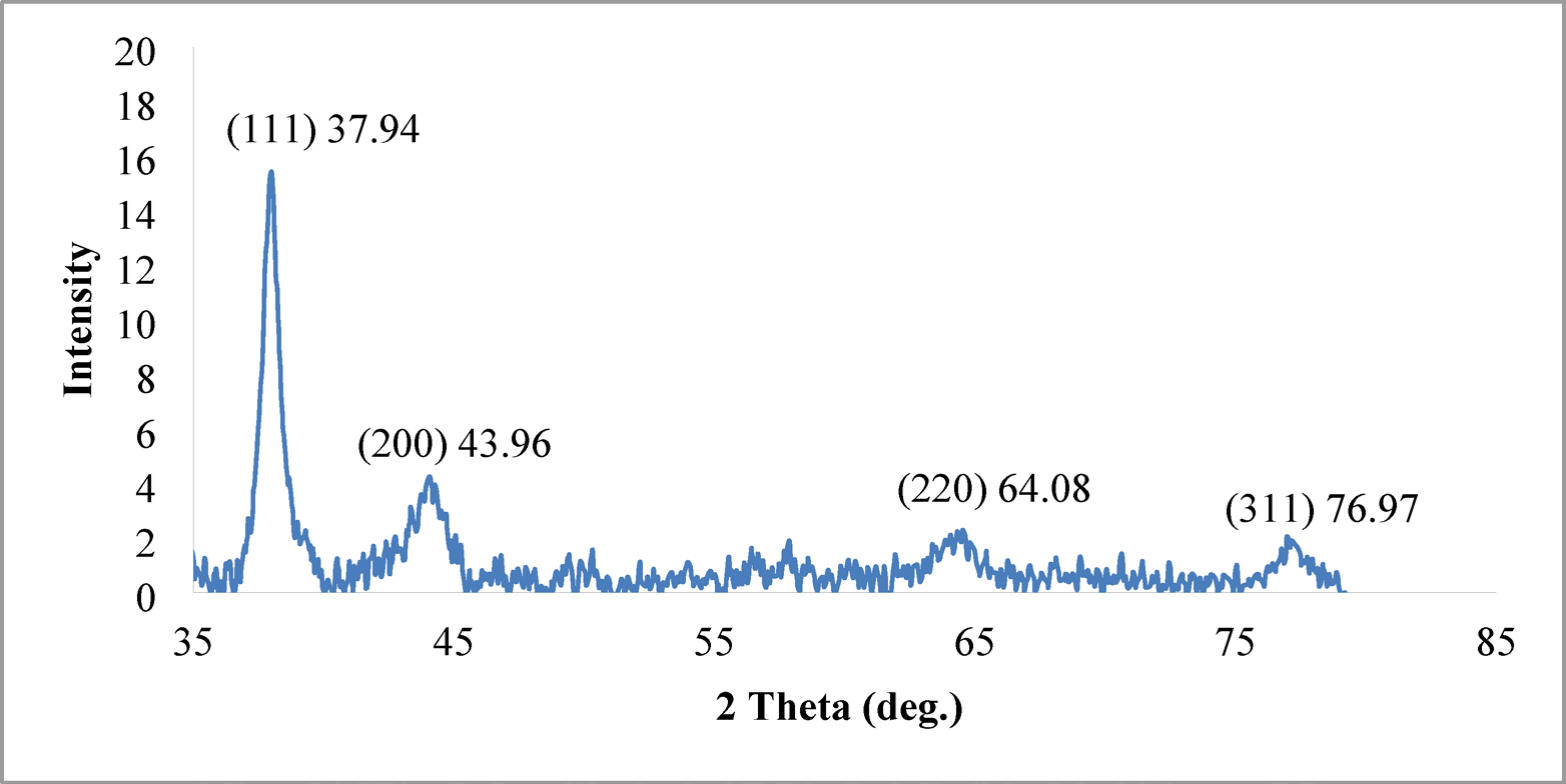
The XRD pattern of the prepared Ag-NPs.

The XRD pattern of the prepared Ag-NPs is represented by Fig. 13. It is obvious that four strong Bragg reflections were appeared at 37.94°, 43.98°, 64.32°, and 76.97° belonging to planes of (111), (200), (220) and (311) respectively which can be indexed according to the facets of face centered cubic crystal structure of silver^57^.

### The potential application of the КC/PVA/PTB hydrogel as wound dressing

#### The percent swelling, gel fraction, and mechanical properties of the КC/PVA/PTB gel treated non-woven cotton fabric

**Table 1:** The percent swelling, gel fraction, and mechanical properties of non-woven cotton fabric samples treated with different КC/PVA/PTB formulations.

КC/PVA weight ratio	Swelling (%)	Gel fraction (%)	Tensile strength (MPa)	Elongation at break (%)	E-Modulus (MPa)	Stiffness (mg)	Pore size (µm)	Porosity (%)	Air permeability (cm^3^/cm^2^.sec)
100/0	208 ± 6.2	89.5 ± 3.01	2.19 ± 0.040	85.62 ± 3.36	6.48 ± 0.18	2047 ± 20.5	68.7433	6.327	206.4 ± 4.06
75/25	237 ± 7.1	87.1 ± 2.60	2.01 ± 0.040	82.03 ± 2.52	5.4 ± 0.17	1637 ± 24.6	82.1566	5.128	218.1 ± 3.91
50/50	293 ± 7.3	85.4 ± 2.60	1.76 ± 0.045	78.15 ± 3.13	3.99 ± 0.16	979 ± 14.7	103.3926	3.881	227.2 ± 5.70

**Fig. 14:**
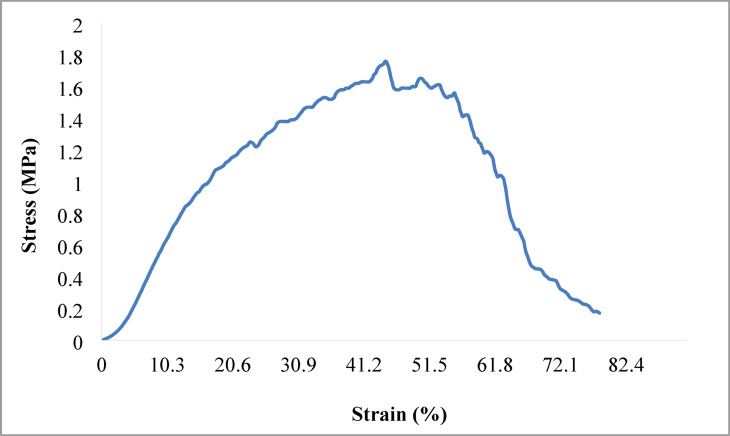
The stress-strain curve of non-woven cotton fabric sample treated with the КC/PVA/PTB formulation.

The percent swelling, gel fraction, and mechanical properties of non-woven cotton fabric samples treated with different КC/PVA/PTB formulations are listed in Table 1. It is clear that decreasing of КC ratio in the КC/PVA blend results in an increasing in percent swelling, pore size, and air permeability along with a decreasing in gel fraction, tensile strength, elongation at break, e-modulus, porosity and stiffness of the КC/PVA dressings which could be associated with the differences between КC and PVA in their chemical structure, molecular weight, rheological properties, film-forming properties, and compatibility with each other^29,30^. Moreover, increasing the pore size along with decreasing the porosity values of treated fabric upon decreasing KC ratio in the КC/PVA blends may be attributed to blocking of the smaller pores, i.e. reduces the porosity percentage, as well as merging the adjacent pathways to create larger, irregularly shaped pathways that ultimately enhances air permeability of the treated fabric as the larger pores offer lower resistance to air flow compared to a dense network of tiny, restrictive pores^58,59^. Furthermore, the mechanical integrity of a wound dressing should be evaluated to ensure that it can resist the physical rigors of the daily wear, without losing its seal, tearing, or causing skin secondary trauma^60^. Figure 14 shows the stress-strain curve of non-woven cotton fabric sample treated with the КC/PVA/PTB formulation prepared at optimum conditions.

##### Antibacterial properties

**Table 2:** The swelling, gel fraction, and antibacterial properties of non-woven cotton fabric samples treated with different КC/PVA/PTB formulations.

КC/PVA formulation type	SW (%)	GF (%)	Reduction (%)	
			S. aureus	E. coli
КC/PVA/PTB	293 ± 7.3	85.4 ± 2.60	95.27 ± 0.95	96.79 ± 0.97
КC/PVA/PTB/curcumin	227 ± 3.5	91.7 ± 2.3	100 ± 1.00	99.36 ± 0.99
КC/PVA/PTB/Ag-NPs	244 ± 6.0	83.5 ± 2.1	100 ± 1.00	100 ± 1.00

PVA/КC weight ratio, 1; [PTB], 4%; [Ag-NPs], 1%; [curcumin], 0.1%.

Table 2 illustrates the impact of treating non-woven cotton fabric samples with the КC/PVA/PTB, КC/PVA/PTB/curcumin or КC/PVA/PTB/Ag-NPs formulations on swelling, gel fraction, as well as antibacterial properties of treated samples. It is well seen that treating of non-woven fabric sample with the КC/PVA/PTB/curcumin formulation gives rise to an enhancement in percent gel fraction along with a decreasing in swelling properties of such sample, compared with that sample treated with the КC/PVA/PTB formulation, i.e. in absence of the curcumin. The enhancement in percent gel fraction of such treated sample may be associated with the upgrading in the cross-linking magnitude inside the КC/PVA/PTB gel structure of that sample resulting from increasing of the H-bonding due to curcumin that in the same time decreases swellability of the treated sample due to its hydrophobic characters^61^. Moreover, treating of fabric sample with the КC/PVA/PTB/Ag-NPs formulation resulted in lowering of both the gel fraction as well as swelling properties of treated sample. It seems that the Ag-NPs impair H-bonding inside the КC/PVA/PTB gel structure causing a partial solubility to that gel^61^. On the other hand, Table 2 reveals that: (i) the fabric sample treated with КC/PVA/PTB formulation have antibacterial activities against both the *S. aureus* (G + ve) and *E. coli* (G-ve) bacteria, reflecting the antibacterial properties of the borate anions^62^, (ii) introducing of the Ag-NPs as a bio-active agent into the КC/PVA/PTB formulation brings about a significant improvement in the antibacterial properties of the treated fabric sample as a direct consequence for:

a) generation of Ag^+^ cations which inactivates the bacterial DNA according to the following Eq.^9^:

1 $$O_{{2\left( {aq} \right)}} + 4H_{3} O^{ + } + 4Ag_{{\left( s \right)}} \to 4Ag_{{\left( {aq} \right)}}^{ + } + 6H_{2} O$$

and/or,

b) oxidation of the bacterial molecular structure due to the formation of oxygen free radicals as represented by the following Eq.^9^:

2 $$H_{2} O + \left( {1/2} \right)O_{2} \xrightarrow{{Ag^{ + } }}H_{2} O_{2} + H_{2} O + \left( O \right)$$

and iii) inclusion of curcumin as a bio-active agent into the КC/PVA/PTB formulation significantly upgrades the antibacterial properties of treated sample due to liberation of the entrapped curcumin as a phenolic compound from the КC/PVA/PBH/curcumin gel matrix^60^.

##### Cytotoxic studies

**Fig. 15:**
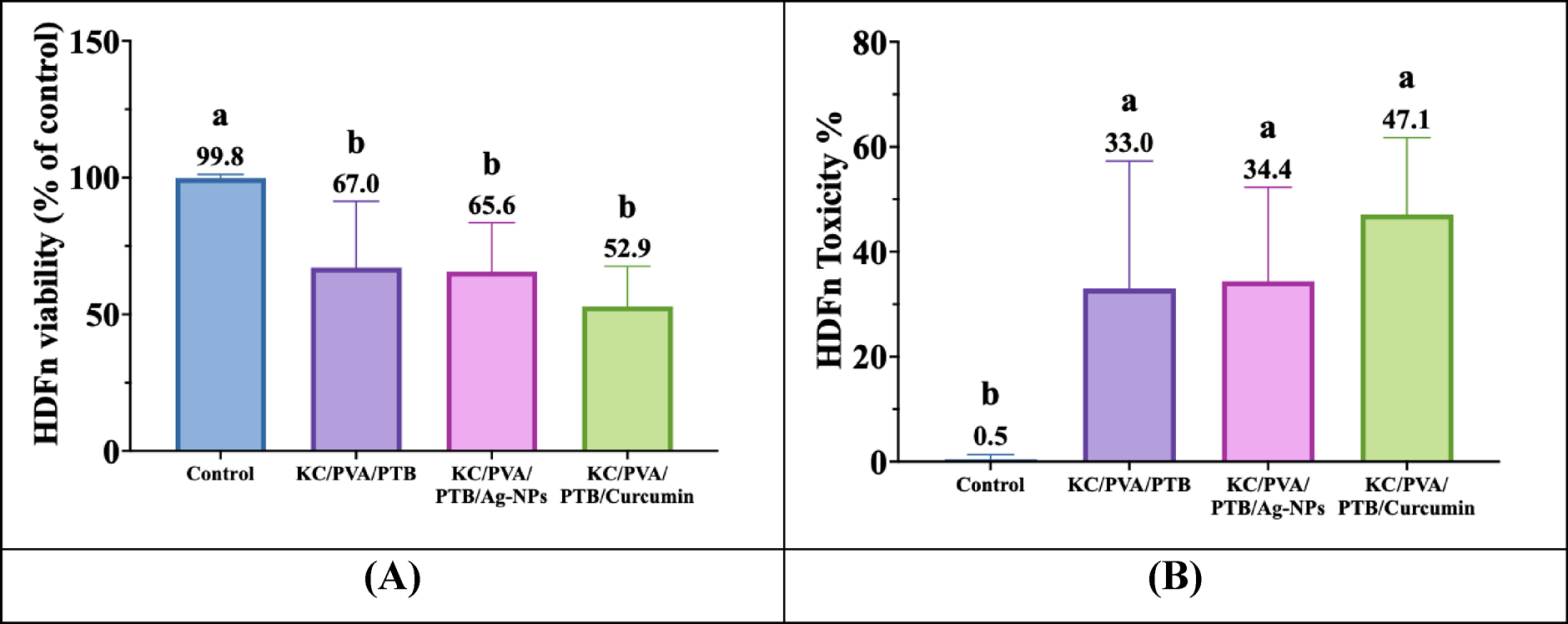
The cell viability of КC/PVA/PTB, КC/PVA/PTB/Ag-NPs, and КC/PVA/PTB/Curcumin on HDFn cell line after 24 h. (i) the cell viability, (ii) the cell toxicity. Using one-way ANOVA with Tukey’s post hoc analysis, the different fabrics differ significantly from the control cells (*p* < 0.05), but no significant differences are observed among them. Data are represented as mean ± SD (*n* = 6). Columns with the same letters are not significantly different.

**Fig. 16:**
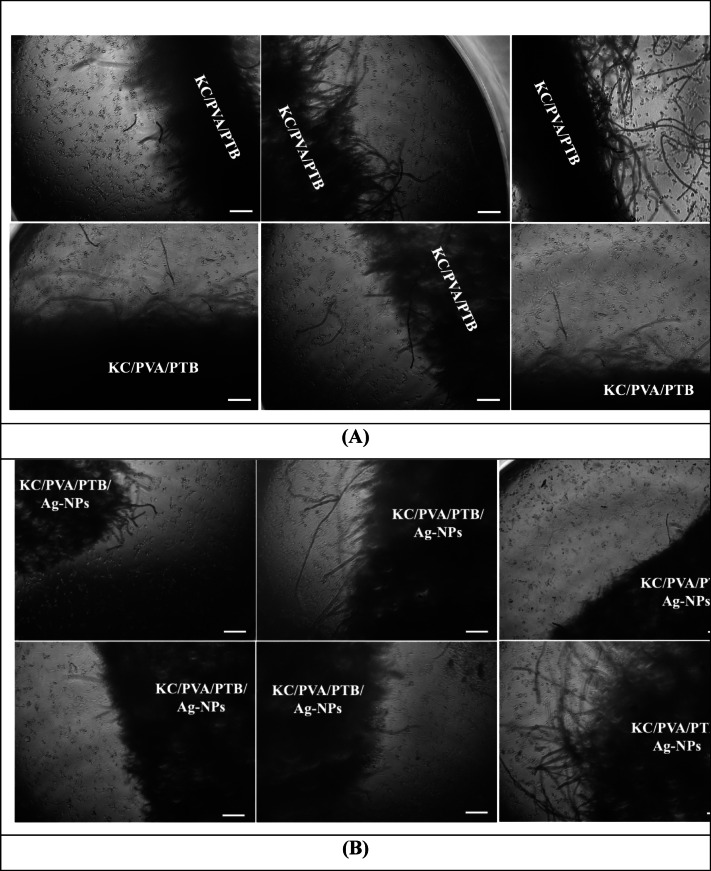

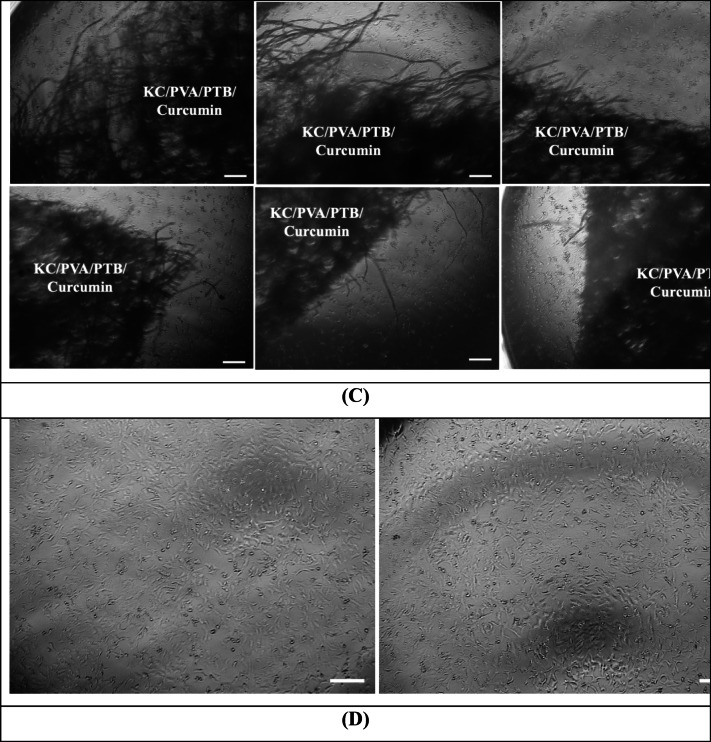
The morphological changes of HDFn cells after 24 h in contact with (A) КC/PVA/PTB, (**B**) КC/PVA/PTB/Ag-NPs, (**C**) КC/PVA/PTB/Curcumin, and (**D**) control cells. The photos show moderate cellular deformities in cells in contact with the fabrics. The scale bar is 100 μm.

The different fabric formulations have been analyzed for their cytotoxic effects on skin fibroblasts after 24 h of incubation. The MTT assay was applied to assess the acute effect of application. Upon applying the different fabrics, the cell viability was significantly decreased compared with the untreated control cells as seen in Fig. 15(A and B). Cell viability decreased in the following order: КC/PVA/PTB, КC/PVA/PTB/Ag-NPs, and КC/PVA/PTB/Curcumin. The cell toxicity ranged from 33% to 47.1%. The morphological behavior of cells in contact with each fabric was illustrated in Fig. 16(A-D). The photos showed moderate deformities in cellular shape and more stressed cells compared with untreated control cells.

##### SEM and EDX

**Fig. 17:**
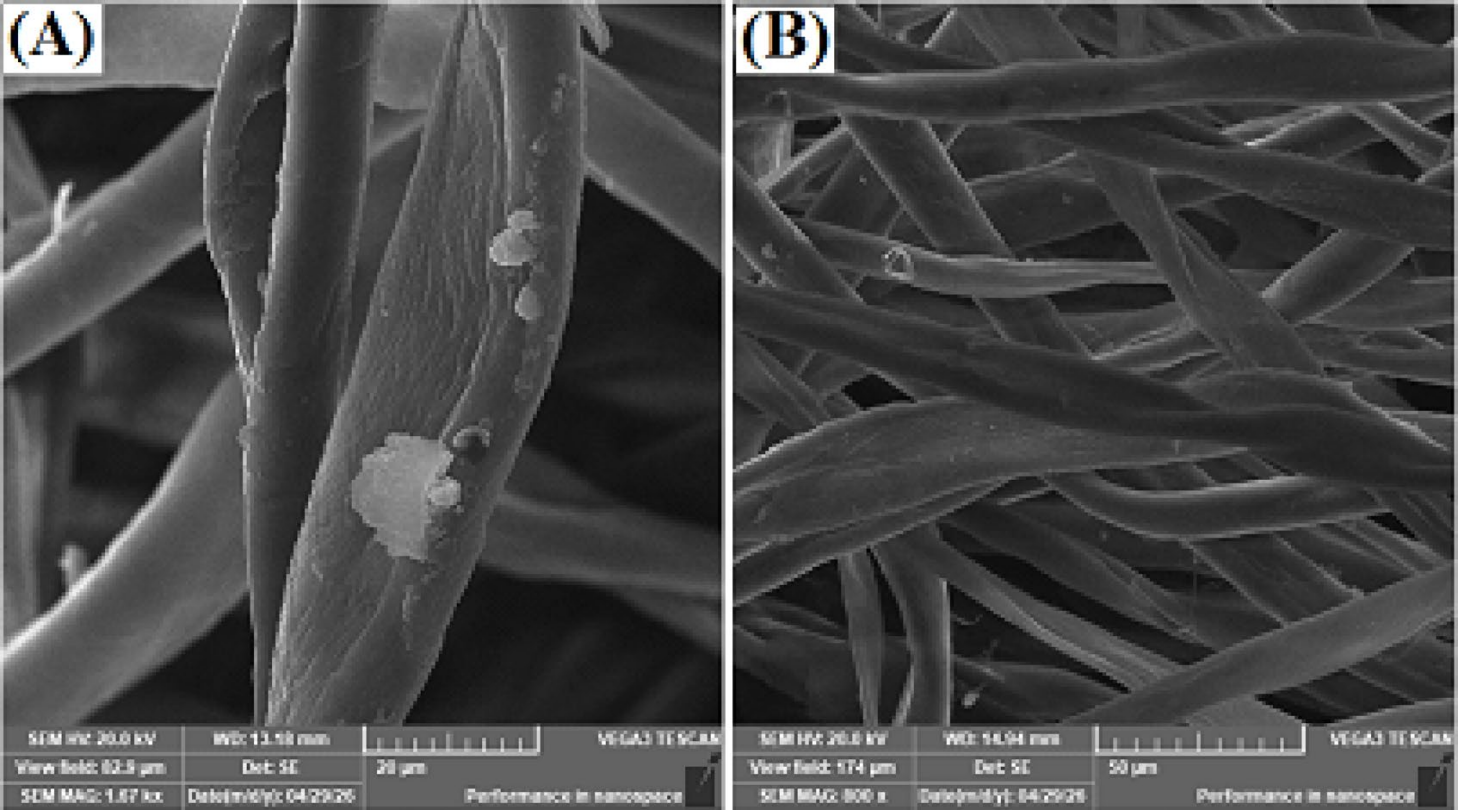
SEM images of non-woven cotton sample treated with the КC/PVA/PTB/Ag-NPs formulation (**A**) and untreated non-woven cotton sample (**B**).

**Fig. 18:**
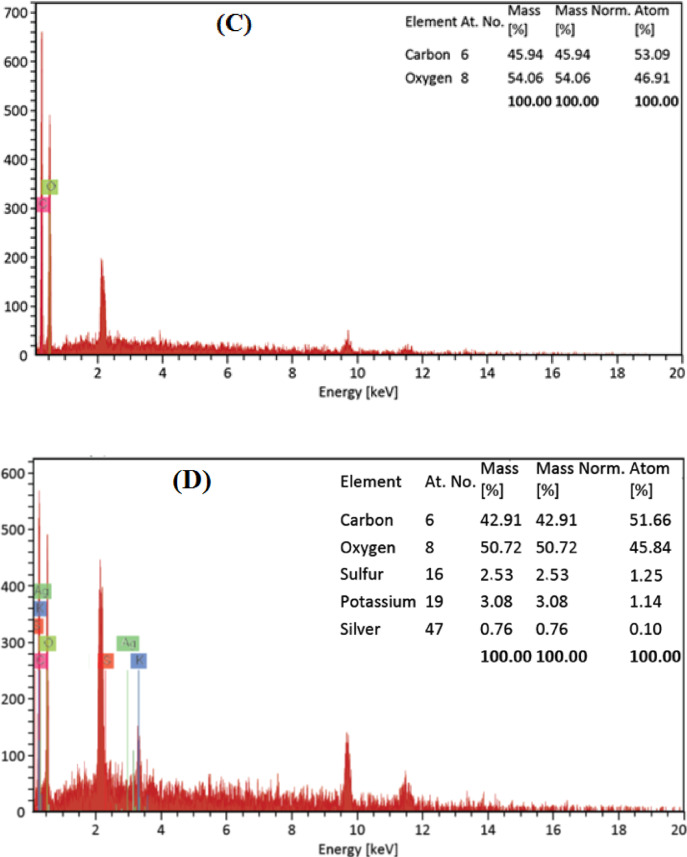
EDX images of untreated non-woven cotton sample (**C**) and non-woven cotton sample treated with the КC/PVA/PTB/Ag-NPs formulation (**D**).

Figure 17 illustrates SEM images of an untreated non-woven cotton sample (A) and non-woven cotton sample treated with the КC/PVA/PTB/Ag-NPs formulation (B). It is well seen from image B that the fabric sample is coated with a homogenous layer of the above mentioned bio-polymers, compared with the untreated sample. On the other hand, Fig. 18 depicts EDX images of an untreated non-woven cotton sample (C) and non-woven cotton sample treated with the КC/PVA/PTB/Ag-NPs formulation (D). It is clearly seen from image D that the coated sample contains the elements carbon, oxygen, sulfur, potassium, and silver whereas the image C contains only the elements carbon and oxygen, the matter that clearly confirms coating of the non-woven cotton fabric with the КC/PVA/PTB/Ag-NPs formulation.

###  A benchmarking table

The results of some of studies that concerned with the KC/PVA hydrogel are summarized and listed in Table 3.

**Table 3:** A benchmarking table.

The most important results	Reference
Different КC/PVA blends were physically crosslinked using four successive freezing–thawing cycles to prepare different КC/PVA hydrogels. The addition of KC to PVA enhances the swelling degree up to 224%, compared to 115% registered to the neat PVA.	^36^
КC/PVA hydrogels was prepared using (3-aminopropyl triethoxysilane as a crosslinker. The hydrogels efficient swelling reached to 200%. The hydrogel samples exhibit strong antibacterial activities against S. aureus and a little against E. coli.	^37^
КC/PVA bio-composite films were prepared for wound care applications. The prepared films exhibited highest mechanical performance expressed in Young’s modulus of 6.25 MPa, tensile strength of 5.65 MPa, and elongation at break of 608.96%. Some of the prepared films showed antibacterial inhibition zones 9 and 10 mm against *S. aureus* and *E. coli*, respectively.	^40^
The prepared КC/PVA/PTB hydrogel exhibits a superior liquid-holding capacity of 1020% and gel fraction of 98.6% while the КC/PVA/PTB formulation treated non-woven cotton fabric as a dressing exhibits superior antibacterial activities as well as significant mechanical properties expressed in good tensile strength, elongation at break, elastic modulus, stiffness, air permeability, and porosity properties.	The current research work

## Conclusions

- The ideal conditions for preparing the КC/PVA gel are: КC/PVA weight ratio, 1; PTB concentration, 4%; and steeping time of the КC/PVA blend in PTB, 8 h.
- At optimum conditions, the liquid-holding capacity of the КC/PVA/PTB hydrogel was 1020% whereas its gel fraction was 98.6%.
- Among boric acid, potassium chloride, borax, and PTB as crosslinkers for the КC/PVA matrix, the PTB achieves the highest crosslinking extent expressed in the superior liquid-holding capacity and gel fraction properties of the formed КC/PVA/PTB gel.
- The chemical structure of the КC/PVA/PTB crosslinked film was confirmed using FTIR analysis.
- Treating non-woven cotton fabric samples with КC/PVA (50/50) matrices containing curcumin or silver nano-particles (Ag-NPs) followed by crosslinking with PTB imparted the treated fabric samples with superior antibacterial activities.
- The prepared Ag-NPs were evaluated via UV–Vis, TEM, and XRD analysis.
- The treated fabric exhibits moderate cytotoxicity.
- The prepared dressings were characterized via SEM.
- The EDX analysis of the Ag-NPs loaded dressing proved the presence of Ag-NPs onto such dressing with Ag - content of 0.94% (w/w).

## Data Availability

Yes; all data supporting the findings of this study are available within the paper and its Supplementary Information.
